# Therapeutic Effects of Photobiomodulation Combined with Exercise on Patients with Peripheral Artery Disease Plus Diabetic Foot Ulcer: A Pilot and Feasibility Study

**DOI:** 10.3390/life15091391

**Published:** 2025-09-01

**Authors:** Shang-Zhen Chen, Tetsuya Takahashi, Hei-Jeng Lai, Hsi-Hsun Su, Yu-Jung Cheng

**Affiliations:** 1 Division of Physical Therapy, Department of Physical Medicine and Rehabilitation, Ministry of Health and Welfare Feng Yuan Hospital, Taichung 42055, Taiwan; s19006024@gmail.com (S.-Z.C.); hueyjen@fyh.mohw.gov.tw (H.-J.L.); lalunesu@gmail.com (H.-H.S.); 2 Department of Physical Therapy, Graduate Institute of Rehabilitation Science, China Medical University, Taichung 406040, Taiwan; 3 Faculty of Health Science, Juntendo University, Tokyo 113-0033, Japan; te-takahashi@juntendo.ac.jp; 4 Department of Rehabilitation, China Medical University Hospital, Taichung 404327, Taiwan

**Keywords:** photobiomodulation therapy, diabetic foot ulcers, peripheral artery disease, exercise

## Abstract

Background: Diabetic foot ulcers (DFUs) in patients with peripheral artery disease (PAD) are difficult to treat and associated with poor healing outcomes. Photobiomodulation therapy (PBMT) and exercise have shown individual benefits, but evidence on their combined effects is limited. Objective: To evaluate whether PBMT combined with resistance exercise improves wound healing and walking ability in patients with DFU and PAD. Methods: In this pilot randomized trial, 11 patients with DFU and PAD were allocated to either PBMT plus supervised exercise or exercise alone for 4 weeks. Outcome measures included wound size, skin temperature, and 6-min walking distance. Results: PBMT combined with exercise improved wound healing and walking capacity compared with baseline; however, no significant between-group differences were observed. A positive correlation was observed between post-PBMT plantar skin temperature and percentage of wound reduction. Conclusions: PBMT combined with resistance exercise may enhance wound healing and functional mobility in patients with DFU and PAD.

## 1. Introduction

Diabetic foot ulcers (DFU) are among the most serious complications of diabetes mellitus (DM) and associated with high risk of infection, amputation, and mortality. The global prevalence of diabetic foot ulcers is 6.3% and is higher in patients with type 2 diabetes (6.4%) than in those with type 1 (5.5%) [1]. More than half of these ulcers become infected [2]. About 20% of serious infections lead to some level of lower-extremity amputation [3]. DFUs can lead not only to the loss of the lower extremity, but also to high mortality and morbidity. According to previous studies, about one-third of DFUs fail to heal [4] and progress to lower-extremity amputation.

DM is a major risk factor for developing peripheral artery disease (PAD) due to its association with atherogenesis [5]. Lower-extremity PAD affects >230 million adults worldwide and is associated with an increased risk of various adverse clinical conditions, such as coronary artery disease and stroke [6]. When PAD complicates DFUs, which occurs in up to 50% of cases, it significantly increases the risk of delayed wound healing and lower-extremity amputation. Moreover, PAD also contributes to reduced mobility, impaired quality of life [7,8], and increased cardiovascular risk, making this comorbidity particularly challenging to manage.

Revascularization reduces the risk of major amputation and improves amputation-free survival in patients with severe PAD [9,10]; however, no single revascularization method has been proven superior [11], and more effective adjunctive interventions are needed for DFUs. Photobiomodulation therapy (PBMT) has emerged as a promising approach [12,13,14,15,16], with evidence showing accelerated ulcer healing through reduced inflammation, enhanced granulation tissue formation, fibroblast and keratinocyte proliferation, and collagen synthesis [17,18]. A meta-analysis by Mendes-Costa et al. reported an average wound closure of −5.25 cm^2^ with PBMT [19], particularly when radiant exposures of 3–7 J/cm^2^ and wavelengths of 630–660 nm or 850–890 nm were applied [20].

Although exercise was not able to facilitate diabetic ulcer healing [21], exercise may prevent muscle loss in diabetic and PAD patients who experience a faster decline in muscle mass and strength [22,23,24,25]. Patients with diabetic PAD showed less walking distance in exercise testing [26], and diabetic PAD patients presented lower physical activity levels and reduced physical function when compared with non-diabetic PAD patients [27]. Resistance exercise was an effective strategy to counteract the deterioration of muscular performance and walking ability in patients with DM [28,29,30] and patients with PAD [31].

We therefore conducted this study to evaluate whether PBMT combined with exercise improves wound healing and walking ability in patients with DFUs and PAD. To our knowledge, no prior randomized controlled trial has examined this specific combination in this patient population, making this study the first to address this gap.

## 2. Materials and Methods

### 2.1. Ethics Statements

This study was conducted in accordance with the Declaration of Helsinki and approved by Feng-Yuan Hospital’s ethical review board (number: 109003). Written informed consent was obtained from all participants.

### 2.2. Study Design and Participants

Participants were recruited from the patients admitted to the vascular surgery departments at Feng-Yuan Hospital, Taichung, Taiwan. Participants were recruited from the vascular surgery departments or referred by the rehabilitation departments between March 2021 and August 2022. All participants were diagnosed based on the following criteria: (1) diagnosis of Type 2 DM; (2) PAD was diagnosed by an angiogram, duplex ultrasonography, or ankle–brachial blood pressure index [32]; (3) the ulcer was located on a lower extremity; (4) the ulcer had persisted for a minimum of two weeks without any signs of healing or with signs of deterioration. Exclusion criteria were as follows: (1) evidence of acute cellulitis and osteomyelitis in the affected extremity; (2) presence of any of one or more medical conditions, including hepatic, hematologic, or immune disease [33]; (3) presence of malignant disease not in remission for more than 5 years; (4) use of oral or parenteral corticosteroids, immunosuppressive, or cytotoxic agents; (5) pregnancy.

**Figure 1:**
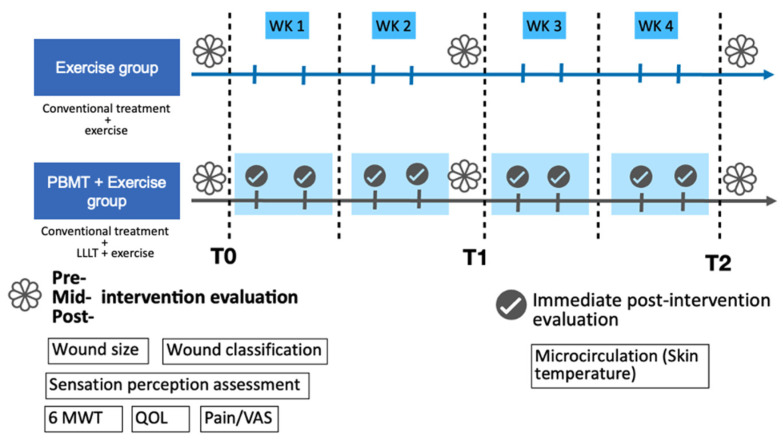
Study design and outcomes measures for each phase of the study. WK, week; LLLT, low-level LASER therapy; 6 MWT, 6-min walking test; QOL, quality of life; VAS, visual analogue scale.

After recruitment and screening for eligibility, the participants were randomized into two groups. Randomization was conducted using a simple, stratified approach. The first participant was assigned to either the intervention or control group using an online random number generator. Subsequently, stratified allocation was performed based on two factors: (1) wound size (≥7 cm^2^ or <7 cm^2^) and (2) ambulatory status (ambulatory vs. non-ambulatory) [34,35]. For each stratum, participants were alternately assigned to the PBMT+exercise group or the exercise-only group to ensure balance. Allocation was performed by the principal investigator at the time of enrollment. The experimental group (PBMT+exercise) underwent PBMT combined with exercise therapy, while the exercise group received exercise therapy alone. Both groups received conventional wound treatment. Details are illustrated in Figure 1.

### 2.3. PBMT Irradiation

Patients were exposed to a light dose of 7.5 mW/cm^2^ on the wound surface. After the wound was cleaned and washed with normal saline, the wound was dried before being exposed to PBMT. A continuous wave from a gallium–aluminum–arsenide (Ga-Al-As) diode low-level LASER (TI-816-20, Transverse industries, Taiwan) with wavelengths of 660 nm and 830 nm irradiated for 10 min, 2 to 5 times per week. Total treatment time was four weeks. After LASER irradiation, the ulcers were dressed with 1% silver sulfadiazine cream, covered with gauze, and bandaged, or treated according to the physician. The attendance rate was recorded as the percentage of attendance per patient.

PBMT was delivered using a diode laser device equipped with a total of 30 diodes,15 at 660 nm and 15 at 880 nm. The system consisted of five modules (six diodes per module) with a total output ≤1500 mW across a ≤200 cm^2^ treatment area, yielding an average power density of ~7.5 mW/cm^2^. Based on device calibration, 10 min of exposure corresponded to ~4.5 J/cm^2^. The dual-wavelength setting (660 nm + 880 nm) was chosen to combine superficial tissue stimulation (red spectrum, promoting epithelial and dermal repair) with deeper penetration effects (near-infrared spectrum, improving vascular and neural function). The selected energy density (4–7 J/cm^2^) and wavelength ranges (red: 630–660 nm; near-infrared: 850–890 nm) are consistent with prior systematic reviews and meta-analyses that have identified these parameters as effective for diabetic foot ulcer healing [19].

### 2.4. Exercise Intervention

The exercise program mainly consisted of resistance exercise and functional activity training. The exercise protocol was individualized because of the participants’ different underlying diseases and different levels of functional abilities. In patients who received bypass surgery, the amount of weight-bearing allowed was determined by the physician. The exercise frequency was twice to 5 times per week and four weeks of treatment.

Resistance exercise: Elastic bands or free weights were used for resistance training, including hip extensor, hip abductor, and knee extensor for lower extremities and lateral pull and biceps curl for upper extremities. The intensity was set at 12–14 RPE (rating of perceived exertion) and each for 1–3 sets of 10–15 repetitions. For participants who were not able to attend the rehabilitation department regularly, home exercise was allowed.

Functional activity training: Functional activity training was implemented if the participants lacked ability. The training followed a progressive sequence of functional activities, starting with rolling, moving to supine to sitting, sitting balance, sitting to standing, standing balance, and stepping, until achieving walking ability.

The exercise programs started at least one week after the participants received bypass surgery and depended on the referral time by the vascular surgery departments. The sequence of PBMT and exercise interventions was randomly assigned for each session.

### 2.5. Outcome Measures

Outcome measurements were performed before (T0), 2 weeks (T1), and 4 weeks (T2) after the intervention, including wound size and classification, skin temperature (microcirculation), pain intensity, sensation perception assessment, walking ability, and quality of life. Skin temperature was measured immediately after PBMT irradiation in the PBMT + exercise group.

Wound size was measured before PBMT treatment. The wounds were photographed using an iPhone. The ulcer area in each photograph was measured using Image J 1.51 software (US National Institutes of Health, Bethesda, MD, USA). Wound classification was based on the Wagner classification [36]. The percentage of wound reduction was also calculated. If the participants had more than one wound, the mean size and percentage of multiple wounds of the same person were calculated and used to run the statistics.

Skin temperature was measured using, a non-invasive and reliable method for evaluating cutaneous microcirculation [37]. This technique has been validated in patients with diabetes/DFU and used to assess peripheral circulatory status [38,39]. Patients were examined by infrared thermography while maintaining a lying position for 10 min. The room temperature was between 20 °C and 25 °C. The humidity was set constantly. The infrared thermal camera was positioned 1 m away from the foot of the patient. The regions of interest were the dorsal and plantar sides of the foot. Thermal images were carried out with handheld thermography (Sonel KT-80, SONEL S.A., Świdnica, Poland) with a spatial resolution of 80 × 80 pixels and thermal sensitivity of 0.08 °C. The accuracy was ±2 °C or ±2%.

After treatment, each patient was evaluated for pain status. A visual analog scale (VAS) score of wound pain was requested and recorded [40].

Protective sensation was assessed using the 10 g Semmes–Weinstein monofilament (SWMN) at standard plantar sites. This monofilament test is widely recommended for screening diabetic peripheral neuropathy and has demonstrated predictive validity for foot ulcer risk in patients with diabetes [41,42,43]. The buckling force for the 5.07 mono-filament is 10 g, which is also the force felt by the patient when the monofilament bends. Loss of protective sensation leads to the inability to sense the 5.07/10 g SWMN. Ten sites of the foot were tested, including the dorsal surface of the foot between the base of the first and second toes, the first, third, and fifth toes, the first, third, and fifth metatarsal heads, the medial and lateral midfoot, and the heel. If the patient was unable to perceive the monofilament at more than 4 of 10 sites, the sensation was regarded as abnormal [44]. The sensitivity and specificity were 93.1% and 100.0%, respectively.

Walking capacity was measured with the 6-min walking test (6MWT) following standard procedures. The 6MWT is a standardized and validated measure of submaximal functional capacity, and in patients with diabetes, it has been shown to be reliable and associated with cycle ergometer graded exercise test [45]. The 6MWT was carried out in a marked corridor 30 m long. The test involves the patient attempting to cover the maximum distance within 6 min. The patient was allowed to walk at their desired speed but not allowed to trot or run. The patient was allowed to stop and rest at any time, and every such situation was recorded. The maximum walking distance was recorded.

QOL was assessed with the SF-36 (36-item Short-Form Health Survey). The SF-36 has been widely used in patients with DM [46] or PAD [47] for surveying their quality of life. The SF-36 scoring system contains 36 items in 8 domains: physical functioning (PF), physical role functioning (RP), emotional role functioning (RE), vitality (VT), mental health (MH), social functioning (SF), bodily pain (BP), and general health (GH) perceptions. Each domain contained 2–10 items. Moreover, the score for each domain was transformed into a scale from 0 to 100. A higher score indicates higher functioning and better health and QOL.

### 2.6. Statistical Analysis

Data obtained from the experiments were expressed as mean ± SD. The statistical analysis was performed with the use of GraphPad Prism software (GraphPad Software, Boston, Massachusetts, USA, version 9). The normality of the data was tested with the Shapiro–Wilk test. For intra-group comparison, significant differences of measurement were analyzed using the Friedman test and Wilcoxon signed-rank test, while between-group comparison was analyzed by the Mann–Whitney test. Category data were analyzed by the Chi-square test. Correlations were calculated by nonparametric Spearman correlation to identify the relationships between the covariates. Statistical significance was set at *p* < 0.05. The missing data of the participants lost to follow-up at T1 were imputed by linear interpolation. These data included the variables of wound size, wound grade, skin temperature, VAS, 6MWT, and scores of the SF-36.

### 2.7. Ethics

This study was conducted in accordance with the Declaration of Helsinki and approved by the Institutional Review Board of Feng-Yuan Hospital (approval number: 109003; date of approval: 25 March 2020). The trial was registered in the Thai Clinical Trials Registry (TCTR20230810003).

## 3. Results

### 3.1. Participants

**Figure 2:**
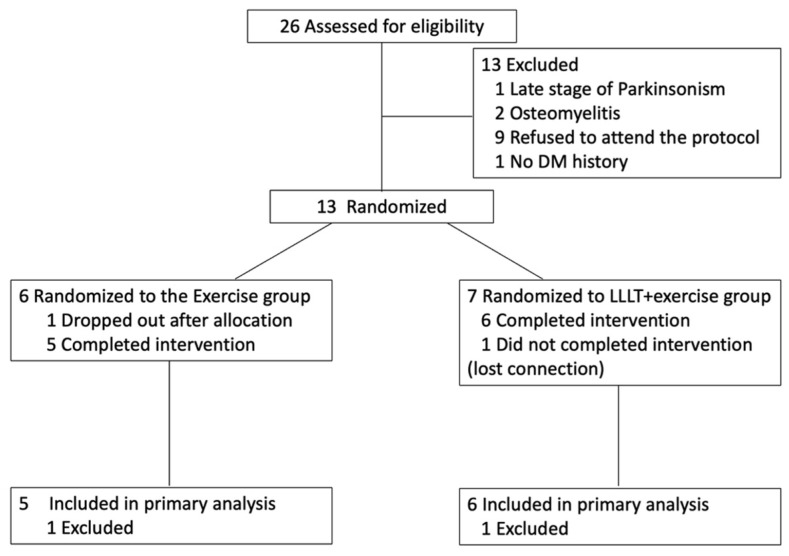
Consort flow diagram of people through interventions. DM, diabetes mellitus; LLLT, low-level LASER therapy.

A total of 26 participants were assessed for eligibility, and 13 participants were recruited (Figure 2). Of these patients, one was lost to follow-up and one dropped out after allocation. Finally, the data from 11 participants were analyzed for this study. Of the six patients who completed the PBMT irradiation, an attendance rate of 100% was defined as attending PBMT sessions at least twice per week for four weeks. The average irradiation frequency per week was 2.46 times/week (range of 2–3.75 times/week). Table 1 reports the characteristics of the participants. There were no significant differences in the baseline characteristics between the two groups (*p* > 0.05).

### 3.2. Clinical Outcomes Before and After Intervention

Baseline and post-intervention outcome measures for both groups are summarized in Table 2 and Table 3. Within-group analysis showed significant improvements in wound size, percentage of wound reduction, and 6MWT distance in the PBMT + exercise group, whereas no significant changes were observed in the exercise-only group. No significant within-group changes were found in skin temperature, and between-group comparisons revealed no significant differences in any outcome, including the SF-36 scores.

### 3.3. Between-Group Differences in Clinical and Functional Outcomes

Between-group comparisons of changes from baseline to week 4 are shown in Table 4 (clinical outcomes) and Table 5 (SF-36 scores). No statistically significant differences were observed in any outcome measure. Effect size analysis indicated large effects for wound size, plantar skin temperature, and pain reduction, and a moderate effect for 6MWT, all favoring PBMT + exercise. By contrast, most SF-36 domains showed small effect sizes, except for the body pain subscale, which demonstrated a large effect favoring PBMT + exercise. These findings suggest potentially meaningful clinical benefits despite the lack of statistical significance, which may be related to the small sample size and variability among participants.

### 3.4. Effects of PBMT on Sensation Perception

Results of the monofilament assessment are shown in Table 6. No significant between-group differences were observed at baseline or after the intervention. Although the number of detectable sites increased in some feet, impaired status persisted in ulcerated legs. The proportion of normal feet increased in both groups by the end of the intervention.

### 3.5. Correlation Between Skin Temperature and Percentage of Wound Reduction

Although skin temperature in the wounded leg did not change significantly between groups, a Spearman’s correlation analysis was conducted to examine the relationship between skin temperature and the percentage of wound reduction. As shown in Table 7, a strong positive correlation was observed between plantar-side skin temperature and wound reduction, particularly at T1 (r_s_ = 0.8857, *p* = 0.0333) and the mean of T1 and T2 (rs = 0.9429, *p* = 0.0167) in the PBMT + exercise group.

## 4. Discussion

To our knowledge, this is the first study to investigate the combined effects of PBMT and resistance exercise in patients with diabetic PAD and foot ulcers. The 4-week intervention significantly improved wound healing and walking capacity and revealed a strong positive correlation between post-PBMT plantar-side skin temperature and the percentage of wound reduction.

PBMT has been reported to accelerate wound healing in DFUs by reducing inflammation, enhancing granulation tissue, and stimulating fibroblast and keratinocyte proliferation, as well as collagen synthesis [12,13,14,15,16,17,18]. Beyond tissue repair, PBMT also promotes angiogenesis and improves microcirculation, effects that are partly mediated through nitric oxide (NO) signaling [38,48,49,50,51,52,53]. Since impairment of NO signaling and endothelial dysfunction are common in patients with PAD and diabetic neuropathy [54,55], these mechanisms are particularly relevant for this population. Taken together, these biological effects are consistent with the improvements in wound healing and walking capacity observed in the PBMT + exercise group in our study.

Notably, the PBMT + exercise group demonstrated a significant improvement in 6-min walking distance, highlighting the potential of this combined intervention to restore mobility in patients with diabetic PAD. Compared to 18 m in the exercise-only group, the PBMT + exercise group gained 30.67 m on average.

While both groups received resistance and functional training, some participants in the control group appeared to rely more on home-based exercise, which may have limited the effectiveness of the intervention. In contrast, participants in the PBMT + exercise group were required to visit the hospital regularly for PBMT sessions, which also increased the likelihood of receiving exercise under supervision. This likely contributed to better adherence and greater training efficacy, but also constitutes a potential source of bias when interpreting the greater improvements observed in the PBMT + exercise group.

Previous studies have shown that supervised exercise improves walking ability more than unsupervised programs [56,57], and that diabetic PAD patients, who often have reduced physical activity and mobility [27], may respond less to exercise alone. According to McDermott et al. [31], the 6MWT of participants with PAD who did not engage in exercise interventions declined over time. In our study, the exercise-only group maintained but did not significantly improve their walking capacity, suggesting that the exercise protocol was sufficient to prevent further decline. However, only the PBMT + exercise group showed significant improvement, which could be attributable to additive benefits of PBMT—physiologically through improved circulation, and behaviorally through enhanced adherence with hospital-based supervision. These findings support the potential of PBMT as a valuable adjunct to exercise in maintaining or restoring functional mobility in patients with diabetic PAD.

Our findings also provide exploratory insights into potential markers and outcomes for future research. Persistently low plantar temperature after PBMT was associated with less wound healing, consistent with the non-thermal mechanism of PBMT [58] and prior reports linking baseline skin temperature with treatment responsiveness [59]. These findings are consistent with plantar temperature serving as an early biomarker of PBMT effects, and with infrared thermography being considered as a tool for monitoring and patient stratification. In addition, effect size analysis revealed a large effect in the SF-36 Body Pain subscale, favoring PBMT + exercise, indicating possible benefits for pain-related quality of life. While preliminary, these observations highlight potentially meaningful clinical signals that merit confirmation in larger, adequately powered trials.

This study has several limitations. First, the sample size was small, which limits statistical power and increases the risk of type II error. Second, although wounds improved significantly within the PBMT + exercise group, no significant differences were found between the groups. The mean PBMT frequency in our study was 2.46 sessions/week (range 2–5), which falls within the range of previous studies (2 sessions/week to daily) [20]. However, lower frequencies may attenuate between-group effects, and Lopez and Brundage [60] reported that daily laser exposure was most effective for wound healing in animal models. Therefore, the relatively low frequency in our study could partly explain the absence of significant between-group improvements. Third, participants in the PBMT + exercise group were required to attend hospital visits for PBMT sessions, which increased the likelihood of receiving supervised exercise. While this may have contributed to better adherence and training efficacy, it also represents a potential source of bias when interpreting the greater improvements observed in this group. Finally, quality of life was assessed using the SF-36, which has been widely applied in patients with diabetes and PAD to capture overall health-related quality of life. However, disease-specific instruments, such as the Diabetic Foot Ulcer Scale–Short Form, may provide greater sensitivity to ulcer-related changes. The choice of the SF-36 was consistent with the feasibility focus of this pilot trial, but future studies could benefit from incorporating disease-specific QoL tools to better capture patient-reported outcomes.

## 5. Conclusions

In conclusion, this pilot study provides preliminary evidence that combining PBMT with resistance exercise enhances wound healing and walking ability in patients with diabetic PAD and foot ulcers. A positive correlation was observed between plantar skin temperature and wound reduction after PBMT, which is consistent with an underlying physiological response related to treatment effects. Although the sample size was small, the findings support the potential of PBMT as a valuable adjunct to exercise-based rehabilitation. Further large-scale studies are warranted to confirm these effects and to explore patient-specific factors influencing responsiveness to PBMT. If validated in larger trials, this combined approach could be integrated into multidisciplinary care models to improve patient outcomes.

## Figures and Tables

**Table 1:** Demographic data.

Variables	Exercise Group (n = 5)	PBMT + Exercise Group (n = 6)	*p* Value
Age (years)	75.60 ± 4.561	72.67 ± 10.84	0.835 ^a^
Gender (M/F)	4/1	3/3	0.303 ^b^
BMI	22.0 ± 2.2	21.8 ± 3.9	0.967 ^a^
Education (years)	6 ± 0	6.6 ± 1.3	>0.999 ^a^
HbA_1_C	1.6 ± 0.5	1.4 ± 0.8	0.469 ^a^
Medical history			
DM duration	16.67 ± 5.77	16.92 ± 10.77	>0.999 ^a^
HTN	4 (80%)	6 (100%)	0.250 ^b^
Stroke	4 (80%)	3 (50%)	0.303 ^b^
MI	1 (20%)	4 (67%)	0.121 ^b^
Dementia	3(60%)	2 (33%)	0.376 ^b^
ESRD	1 (20%)	2 (33%)	0.621 ^b^
Retinopathy	4 (80%)	4 (66%)	0.621 ^b^
Revascularization (Bypass/PTA/None)	5/0/0	4/1/1	0.361 ^b^
Cigarette use			
Never/Past/Current smoker	2/2/1	3/3/0	0.516 ^b^
HBOT therapy	4 (80%)	5 (83%)	0.886 ^b^

Data are expressed as mean ± SD or N (%); ^a^ Mann–Whitney tests; ^b^ Chi-square tests. BMI: body mass index; HTN: hypertension; MI: myocardial infarction; ESRD: end-stage renal disease; PTA: percutaneous transluminal angioplasty; HBOT: hyperbaric oxygen therapy.

**Table 2:** Baseline/post-intervention comparisons in outcome measures within and between the groups.

	Exercise Group (n = 5)			*p* Value ^a^	PBMT + Exercise Group (n = 6)			*p* Value ^a^
	T0	T1	T2		T0	T1	T2	
Wound size (cm^2^)	16.85 ± 20.10	14.39 ± 18.67	11.66 ± 15.47	0.182	5.15 ± 4.45 **^#^**	4.20 ± 3.21	3.35 ± 3.22 **^#^**	0.006 *
% Wound reduction	0 ± 0	11.50 ± 41.87	21.16 ± 59.40	0.182	0 ± 0 **^#^**	11.47 ± 16.71	35.36 ± 18.76 **^#^**	0.006 *
Wound grade	1.6 ± 0.55	1.5 ± 0.58	1.6 ± 0.55	>0.999	1.4 ± 0.80	1.4 ± 0.80	1.4 ± 0.80	>0.999
Skin temperature (°C)								
Plantar side	32.9 ± 2.5	30.1 ± 2.9	31.1 ± 2.8	0.093	28.1 ± 2.2	29.4 ± 2.6	29.5 ± 1.1	0.241
Dorsal side	34.0 ± 2.4	30.8 ± 3.5	32.0 ± 3.0	0.953	30.6 ± 2.7	31.6 ± 3.2	31.7 ± 3.5	0.570
Wound pain (VAS)	3.3 ± 4.2	2.8 ± 2.6	6.7 ± 2.9	0.666	3.2 ± 3.2	3.0 ± 3.1	1.7 ± 2.3	0.801
6MWT (m)	19.67 ± 17.39	19.50 ± 24.14	37.67 ± 34.27	0.500	40.00 ± 36.99	54.17 ± 42.13	70.67 ± 58.12 **^#^**	0.001 *

Values are mean ± standard deviation. 6MWT: 6-min walking test; PBMT: low-level laser therapy; ^a^: baseline and post-intervention comparison within the group by Friedman test, * statistically significant *p* < 0.05; ^#^: post hoc significant between T0 and T2.

**Table 3:** Baseline/post-intervention comparisons in SF-36 within the groups.

	Exercise Group (n = 5)			*p* Value ^a^	Exercise + PBMT Group (n = 6)			*p* Value ^a^
	T0	T1	T2		T0	T1	T2	
SF-36								
Physical function (PF)	3 ± 4.47	1.000 ± 2.236	1 ± 2.24	>0.9999	12.50 ± 13.69	9.167 ± 15.30	10.83 ± 17.44	0.3086
Role: Physical (RP)	0 ± 0	0 ± 0	0 ± 0	>0.9999	0 ± 0	0 ± 0	4.167 ± 10.21	>0.9999
Role: Emotional (RE)	0 ± 0	0 ± 0	0 ± 0	>0.9999	16.67 ± 40.82	33.33 ± 51.64	33.33 ± 51.64	>0.9999
Vitality (VT)	20 ± 17.80	26.00 ± 8.216	25 ± 7.91	>0.9999	33.75 ± 8.329	34.17 ± 18.00	43.33 ± 20.90	0.1687
Mental health (MH)	29 ± 19.4	35.10 ± 9.182	37 ± 10.05	0.5185	48.67 ± 22.26	47.83 ± 28.46	56.33 ± 30.26	0.4527
Social functioning (SF)	25 ± 23.39	17.50 ± 16.77	35 ± 27.10	0.7469	41.67 ± 15.14	45.83 ± 21.89	39.58 ± 21.53	0.8724
Bodily pain (BP)	33.5 ± 32.04	29.00 ± 24.60	22 ± 17.80	0.5185	37.08 ± 7.486	40.42 ± 22.22	53.33 ± 32.62	0.1296
General health (GH)	27 ± 20.19	36.00 ± 19.49	37 ± 21.39	>0.9999	42.50 ± 14.05	38.33 ± 21.60	43.33 ± 17.80	0.1481

^a^: Baseline and post-intervention comparison within groups by Friedman test.

**Table 4:** Changes in outcome measures (T2–T0) between the exercise and PBMT + exercise groups.

	Exercise Group (n = 5)	PBMT + Exercise Group (n = 6)	*p* Value ^a^	Cohen’s d Effect Size
	Differences from T0 to T2	Differences from T0 to T2		
Wound size (cm^2^)	5.197 ± 5.218	1.795 ± 2.022	0.329	0.8597
% Wound reduction	0.962 ± 106.8	35.36 ± 18.76	0.9307	0.4486
Wound grade	0 ± 0	0 ± 0	>0.9999	NA
Skin temperature (°C)				
Plantar side (°C)	−1.740 ± 4.329	1.367 ± 2.353	0.5368	0.8917
Dorsal side (°C)	−1.980 ± 4.906	1.017 ± 2.676	0.5368	0.7584
VAS	2.000 ± 4.950	−1.500 ± 4.231	0.4242	0.7601
6MWT (m)	18.00 ± 20.95	30.67 ± 28.07	0.5952	0.5115

^a^: Comparison of differences between groups by Mann–Whitney U-test. Effect sizes are reported as Cohen’s *d*. Interpretation: small (0.2), medium (0.5), large (0.8). NA: Effect size not applicable due to zero variance.

**Table 5:** Between-group differences in SF-36 scores from baseline (T0) to week 4 (T2).

	Exercise Group (n = 5)	PBMT + Exercise Group (n = 6)	*p* Value ^a^	Cohen’s *d* Effect Size
	Differences from T0 to T2	Differences from T0 to T2		
SF-36				
Physical function (PF)	−2.000 ± 4.472	−1.667 ± 6.055	0.6537	0.062
Role: Physical (RP)	0 ± 0	4.167 ± 10.21	>0.9999	NA
Role: Emotional (RE)	0 ± 0	16.67 ± 40.82	>0.9999	NA
Vitality (VT)	3.000 ± 21.10	9.583 ± 25.12	0.6277	0.283
Mental health (MH)	5.000 ± 20.22	7.667 ± 24.74	0.6970	0.118
Social functioning (SF)	10.00 ± 33.54	−2.083 ± 25.52	0.6450	0.405
Bodily pain (BP)	−11.50 ± 25.16	16.25 ± 27.33	0.1450	1.056
General health (GH)	10.00 ± 33.35	0.8333 ± 11.14	0.9502	0.368

^a^: Comparison of differences between groups by Mann–Whitney U-test. Effect sizes are reported as Cohen’s *d*. Interpretation: small (0.2), medium (0.5), large (0.8). NA: effect size not applicable due to zero variance.

**Table 6:** Baseline/post-intervention comparisons in sensation perception assessment within and between the groups.

		T0			T1				T2			
		Exercise	PBMT + Exercise	*p* Value ^a^	Exercise	PBMT + Exercise	*p* Value ^a^	Exercise	PBMT + Exercise	*p* Value ^a^	*p* Value ^b^	*p* Value ^c^
Wound leg	Normal	2	1		2	1		2	1			
	Abnormal *	1	4	0.1869	1	4	0.1869	1	4	0.1869	>0.9999	>0.9999
Unaffected leg ^‡^	Normal	1	2		1	3		2	3			
	Abnormal *	2	2	0.6592	2	1	0.8091	1	1	0.8091	0.6376	0.6873
Total leg ^#^	Normal	3	3		3	4		4	4			
	Abnormal *	3	6	0.5186	3	5	0.8327	2	5	0.8327	0.7985	0.8578

^‡^ Unaffected leg was defined as the leg without wounds; * abnormal was defined as patients unable to perceive the monofilament at more 4 out of 10 sites; ^#^ the total number of legs was 13, with 6 legs in the control group and 7 legs in the PBMT group; ^a^: comparison between the control and PBMT group by Chi-square tests; ^b^: baseline and post-intervention comparison within the control group by Chi-square test; ^c^: baseline and post-intervention comparison within the PBMT group by Chi-square test.

**Table 7:** The correlation between skin temperature of wounded leg and % wound reduction in the PBMT group.

	T0		T1		T2		Mean of T1 and T2		Mean of T0, T1, and T2	
	r_s_	*p* Value	r_s_	*p* Value	r_s_	*p* Value	r_s_	*p* Value	r_s_	*p* Value
PBMT + exercise group										
Dorsal side	0.08571 ^a^	0.9194	0.4286 ^a^	0.4194	0.4286 ^a^	0.4194	0.4286 ^a^	0.4194	0.3714 ^a^	0.4972
Plantar side	0.1429 ^a^	0.8028	0.8857 ^a^	0.0333 *	0.6000 ^a^	0.2417	0.9429 ^a^	0.0167 *	0.8286 ^a^	0.0583

Number of XY pairs was 6; ^a^ calculated by nonparametric Spearman correlation; * statistically significant, *p* < 0.05.

## Data Availability

The original contributions presented in this study are included in the article. Further inquiries can be directed to the corresponding author.

## Abbreviations

The following abbreviations are used in this manuscript:

PBMT	Photobiomodulation
DM	Diabetes mellitus
PAD	Peripheral artery disease
DFU	Diabetic foot ulcer
6MWT	6-min walking test
